# Editorial: Epigenetic Variation Influences on Livestock Production and Disease Traits

**DOI:** 10.3389/fgene.2022.942747

**Published:** 2022-06-15

**Authors:** Eveline M. Ibeagha-Awemu, Hélène Kiefer, Stephanie McKay, George E. Liu

**Affiliations:** ^1^ Sherbrooke Research and Development Centre, Agriculture and Agri-Food Canada, Sherbrooke, QC, Canada; ^2^ Université Paris-Saclay, UVSQ, INRAE, BREED, Jouy-en-Josas, France; ^3^ Ecole Nationale Vétérinaire d’Alfort, BREED, Maisons-Alfort, France; ^4^ Department of Animal and Veterinary Sciences, University of Vermont, Burlington, VT, United States; ^5^ Animal Genomics and Improvement Laboratory, Agricultural Research Service, United States Department of Agriculture, Beltsville, MD, United States

**Keywords:** livestock, DNA/RNA methylation, histone tail modifications, production, reproduction, nutrition, pathogens/health, stress

The epigenome source of variation modulates gene expression without involving changes to the DNA sequence and is an underutilized source of information that may contribute to improved disease management, reproduction, productivity, and environmental adaptation of livestock. The epigenome which comprises DNA methylation, histone tail modifications, chromatin remodeling, and non-coding RNA species that can transmit epigenetic information (e.g. microRNAs and long non-coding RNAs, etc.) responds to environmental factors (nutrition, pathogens, and climate, etc.) to influence the expression of genes and the emergence of specific phenotypes. Increasing evidence indicates that phenotypic expression results from multi-level interactions between the genome, epigenome, environmental factors, and other non-genetic factors. Furthermore, numerous lines of evidence suggest the influence of epigenome variation on livestock production (e.g. milk production), disease traits, reproduction, and environmental adaptation. Given that livestock breeding depends heavily on the interaction between host genetics, environment (e.g. cold or warm climate, nutrition, pathogens, etc.), physiology (e.g. age) and management practices (e.g. type of feed material, form/quantity of feed), many of which impact the epigenome, it is imperative that attention should be given to epigenome characterization and application in livestock breeding and disease management. Therefore, the aim of this Research Topic is to collect research data on the influences of epigenome alterations (DNA methylation, RNA methylation, histone tail modifications and chromatin remodeling) on livestock production traits, to facilitate gainful use of this important source of variation to support continued improvement in livestock traits and disease management. Non-coding RNA data on livestock is already covered by other Research Topics and is excluded from this topic.

This Research Topic collates four review articles and 19 original research articles covering major topics in epigenetics (DNA methylation, RNA methylation, and histone modification) in relation to livestock health, reproduction, production and response to stress and environmental perturbations in many livestock species, including cattle (seven articles), chicken (five articles), sheep (three articles), pig (two articles), yak (one article), and pearl oyster (one article). Although, many livestock species and a broad range of epigenomics alterations are covered, there is a bias towards DNA methylation alterations and more concentration on cattle, highlighting the need to widen/intensify research efforts to include all livestock species as well as address more issues of health and productivity, which is vital for the effective exploitation of epigenetic information for livestock trait improvement and health management.

The review articles critically examine available epigenetics research data and impacts on livestock health, productivity, reproduction, stress adaptation, and reprograming during somatic cell nuclear transfer. Wang and Ibeagha-Awemu (Wang and Ibeagha-Awemu) present the recent discoveries and evidence on how the epigenetic processes due to DNA methylation, n^6^ methyladenosine (m^6^A) RNA methylation, histone modification, and chromatin remodeling impacts the health and productivity of farm animals, particularly cattle, pig, goat, sheep, and poultry. The investigations collated, examined DNA-methylation alterations mostly at the genome-wide scale and to a lesser extent at specific gene regions and addressed animal response to environmental stressors, nutrition, disease pathogens, and developmental processes. They further discuss the application of epigenetics data in livestock health and production and present gaps in livestock epigenetics research. Given increasing evidence that supports the concept that some acquired traits result from environmental exposure during early embryonic and fetal development (i.e., fetal programming), and which can be stamped in the germline as epigenetic information and transmitted to subsequent generations, Zhu et al., discuss the effects of parental environmental exposures on the epigenetics (DNA methylation, histone modification, chromatin remodeling, and non-coding RNAs regulation) of gametes and the early embryo, and available evidence for transgenerational ineritance in livestock (Zhu et al.). In the same light, Wu and Sirard (Wu and Sirard) present available information on the parental non-genetic effects retained in gametes on the development of the early embryo of cows (dairy), paying particular attention to the metabolism of the mother at the time of conception, *in vitro* culture conditions, and parental age relative to puberty. Examining an assisted reproduction technology, somatic cell nuclear transfer, that is commonly used for the production of cloned animals but with the limitation of low cloning efficiency, Wang et al. presents available data proving that incomplete epigenetic reprogramming contributes to the low developmental potential of cloned embryos, and also describes the regulation of epigenetic reprogramming by long non-coding RNAs (Wang et al.).

A majority of the original research articles (65%) investigate how DNA methylation or m^6^A RNA methylation alterations play essential roles in the regulation of various livestock traits. Two articles present DNA methylation alterations in response to disease pathogens in cattle. Wang et al. present evidence of significant DNA methylation alterations in mammary gland quarters of Chinese Holsteins challenged with *Staphylococcus aureus* compared with non-challenged quarters of the same animals, as well as significant enrichment of several differentially methylated and expressed genes with immune functions (e.g. *IL6R*, *TNF*, *BTK*, *IL1R2*, and *TNFSF8*) in several immune-related pathways indicative of their involvement in cow’s response to *Staphylococcus aureus* infection (Wang et al.). Meanwhile, the role of *Mycobacterium avium subsp paratuberculosis* (MAP) infection on the epigenetic modeling of gut immunity during the progression of Johne’s Disease (JD) is presented with evidence of tissue-specific responses to MAP infection with more DNA methylation alterations (more differentially methylated cytosines and differentially methylated regions) in the ileum lymph node tissue than in the ileum tissue of cows with subclinical JD (Ibeagha-Awemu et al.). Five articles discuss the effects of different stressors or environment change on DNA methylation patterns in livestock, such as differential DNA methylation changes due to the effect of different incubation temperatures and CO_2_ levels in chicken cardiac muscle (Corbett et al.); or due to weaning stress in piglet peripheral blood mononuclear cells (Corbett et al.); or due to heat stress in cattle (Del Corvo et al.); or due to long term stress in chicken red blood cells (Pértille et al.); or due to water depth variation (environment change) in pearl oyster (*Pinctada margaritifera* var. *cumingii*) resulting in shell color variation (Stenger et al.). With regards to product quality, Zhao et al. present the impact of DNA methylation alterations on beef tenderness as well as novel epigenetic data associated with beef quality and further insights into meat science and muscle biology (Zhao et al.). DNA methylation alterations known to influence reproduction traits is the subject of three articles. Perrier et al. report the effects of early life plane of nutrition of Holstein calves on sperm DNA methylation patterns post-puberty (Perrier et al.); while Khezri et al. examine the effect of age and sperm cell condition (fresh or frozen-thawed), demonstrating a significant decrease in the percentage of DNA fragmented sperm cells, and more sperm DNA methylation with increasing age of the bull at differentially methylated regions found between 14 and 17 months of age (Khezri et al.). Examining sheep reproduction traits, Yao et al. demonstrate significant DNA methylation and gene expression changes between high prolificacy (FecBB) and low prolificacy (FecB+) Hu sheep, thereby demonstrating that DNA methylation influences prolificacy capacity by modulating gene expression in the ovaries (Yao et al.). Deviating from a focus on single DNA sites/regions or tissues, Hazard and colleagues show that global DNA methylation rate (GDMR) in blood varies among animals, but is significantly higher in female lambs compared to male lambs, and is moderately heritable and associates significantly with quantitative trait loci harboring genes with known active roles in gene expression (Hazard et al.). Meanwhile, Drouilhet et al. demonstrate significant GDMR variability in sheep blood due to sampling date and breed, and between blood and various tissues (12 somatic tissues and 6 reproductive tissues) (Drouilhet et al.).

In addition to presenting the effects of DNA methylation, the effect of RNA methylation, especially m^6^A RNA methylation, also known to participate in post-transcriptional gene regulation, is discussed. Combining methylated RNA immunoprecipitation sequencing (MeRIP-seq) and RNA sequencing (RNA-Seq) data, Zhang and colleagues show that genes with up-regulated m^6^A peaks but with decreased expression, are mainly enriched in the Wnt signaling pathway, suggesting that this pathway may be modified by m^6^A methylation in *Clostridium perfringens* beta2 toxin-induced porcine intestinal epithelial (IPEC-J2) cells (Zhang et al.). Similarly, Zhang et al. present a positive association between m^6^A methylation abundance (MeRIP-seq) and levels of gene expression (RNA-Seq), as well as a vital role of m^6^A methylation in the modulation of gene expression during yak adipocyte differentiation (Zhang et al.).

The effects of histone modifications, the second most studied epigenetic mechanism, is presented in relation to heat stress, disease/disease pathogens and thermal manipulation. Addressing two active histone modifications, H3K4me3 (predominantly marks active promoters) and H3K27ac (marks both active promoters and enhancers), Chanthavixay et al. present higher differences in histone modifications in the bursal of Leghorns compared to Fayoumis chickens in response to treatment with Newcastle disease virus while under chronic heat stress (Chanthavixay et al.), while David and colleagues show that histone marks **(**H3K4me3 and H3K27me3) known to contribute to environmental memory in eukaryotes, presents higher numbers of differential peaks in the hypothalamus of 35 days old (slaughter age) chickens with implicated peaks found on genes with metabolic, neurodevelopmental, and gene regulation functions, but fewer differences between peaks of the histone marks in muscle tissue in response to thermal stress during embryogenesis (David et al.). Examining liver tissue between healthy and fatty liver hemorrhagic syndrome (FLHS) chickens, Zhu et al. present H3K27ac differential peaks association with multiple known FLHS risk genes with roles in the immune response, and lipid and energy metabolism (Zhu et al.). On a more general note, Zheng et al. present transcriptional changes and the taxonomic status of a new Chinese native cattle known as Wandong (Zheng et al.)

These reports unequivocally present evidence of the involvement of epigenetic marks in defining livestock trait expression and their importance in modulating economically important livestock traits, products, and environmental adaptation. Concerted efforts and more targeted epigenetic research in livestock spanning the exposome (nutrition, disease pathogens, microbiota, chemicals, pollutants, and environmental stress [heat, cold, and climate chane], parental stress, maternal behavior and management practices), species, breeds (genetic variation), and under various conditions will provide a holistic view and understanding of the epigenetic influence on phenotypic variation in livestock production, reproduction, health and environmental adaptation, and gainful exploitation for production and health management.
